# A clinically informed automated evaluation pipeline for medical image segmentation based on Medical Similarity Index

**DOI:** 10.1016/j.phro.2026.100950

**Published:** 2026-03-17

**Authors:** Szuzina Fazekas, Bettina K. Budai, Viktor Bérczi, Pál Maurovich Horvat, Zsolt Vizi

**Affiliations:** a Semmelweis University, Medical Imaging Centre, Üllői str. 78a., 1085 Budapest, Hungary; b University of Szeged, Bolyai Institute, Aradi vértanúk tere 1., 6720 Szeged, Hungary; c Heidelberg University Hospital, Department of Diagnostic and Interventional Radiology, Im Neuenheimer Feld 130.3., 69120 Heidelberg, Germany

**Keywords:** Image segmentation, Evaluation metrics, Image processing pipeline, Relevant metrics, Medical Similarity Index

## Abstract

Accurate tissue delineation is essential in radiotherapy; however, conventional segmentation metrics mainly quantify geometric overlap and lack clinical interpretability. We proposed an automated Python-based evaluation pipeline using a bidirectional local distance-based metric that pairs test and reference contour points after center-of-mass correction and computes a similarity score from averaged Euclidean distances. The framework supports multislice images, multiple masks per slice, and concave mask separation, with open-source code provided. The method was demonstrated on fibroid and prostate MRI datasets, using 233 training cases and 12 test cases. In test examples, overlap scores exceeded 0.90, while Medical Similarity Index scores decreased to approximately 0.40.

## Highlights

• Traditional overlap metrics can miss segmentation errors critical for radiotherapy planning.
• A Python-based pipeline enables clinically informed evaluation of image segmentations.
• The framework was demonstrated on prostate and fibroid magnetic resonance images.
• Inner and outer contour deviations are penalized separately to reflect clinical priorities.
• High Dice (>0.9) can coexist with low Medical Similarity Index (∼0.4) in critical cases.

## Graphical abstract

**Figure:**
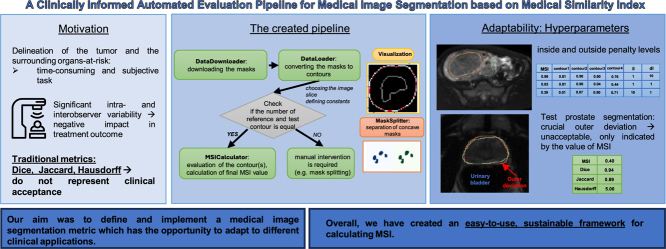

## 1 Introduction

Medical image segmentation plays a crucial role in numerous clinical applications. Modern radiotherapy enables the precise delivery of high radiation doses to the target volume while minimizing exposure to surrounding healthy tissues. Accurate delineation of both the tumor and surrounding organs-at-risk is essential; however, this remains a very time-consuming and subjective task [1], leading to significant variability among clinicians. Moreover, uncertainty and inconsistency in target volume delineation account for the largest errors throughout the radiotherapy workflow, from treatment planning to delivery [2]. The quality of the radiation protocol has been shown to heavily influence patient outcomes. Deficiencies in the treatment plan are reported to result in significantly reduced survival, with head and neck cancer patients experiencing a two-year decrease in overall survival [3]. Significant intra- and interobserver variability in the segmentation of target volumes in breast cancer has been associated with a negative impact on treatment outcome [4]. Accurate brain tumor segmentations are also vital, as these have a direct impact on surgical planning [5]. Prostate segmentation is important not only for radiotherapy planning but also for monitoring prostate volume during disease progression, facilitating multimodal image registration, defining regions of interest for computer-aided diagnosis (CAD), and supporting prostate cancer staging [6]. However, accurate segmentation remains challenging due to the complex anatomy of the prostate and variability caused by changes in prostate morphology [7].

In response to these challenges, an increasing number of automated segmentation methods have been developed (including transformer- [8], diffusion- [9], and Mamba-based models [10]). In radiology, many artificial intelligence-based solutions are now in clinical practice [11]. However, evaluating such algorithms remains inconsistent due to the lack of standardized assessment protocols.

Low correlation between segmentation metrics and dosimetric changes for organs-at-risk (OARs) was shown in brain tumor patients [12]. Poel et al. investigated 23 metrics, including similarity measures (Dice, Jaccard, area under the curve [AUC], etc.), distance measures (Hausdorff, etc.), and classical measures (sensitivity, specificity, etc.). They found that all metrics have limited predictive value for treatment quality and, consequently, suggested revisions toward clinically oriented metrics.

This study addressed these issues by implementing an adaptable segmentation metric that tailors evaluation criteria to different application scenarios. Unlike traditional metrics focused on geometric differences, this approach facilitates task-specific analysis and, through selected examples, demonstrates how the metric highlights the priorities of each imaging context. This flexibility provides a practical and nuanced framework for analyzing automated segmentation beyond geometric agreement.

## 2 Materials and methods

For reproducibility, a detailed description of the methods was provided (S1, S2), along with the GitHub repository [13].

### 2.1 Datasets

For a detailed explanation of the datasets, see Supplementary Materials S3.

We retrospectively identified 161 patients who underwent uterine artery embolization between 2016 and 2020; 31 were excluded due to corrupted data, non-contourable fibroids, or adenomyosis. T2-weighted images were manually segmented in 3D Slicer by a radiology resident and validated by two senior radiologists (with corrections made by consensus). Of these, 124 cases were used for training and 6 for testing, stratified by difficulty.

An open-access multi-site prostate MRI dataset comprising 115 T2-weighted scans with corresponding expert segmentations from three public collections (NCI-ISBI 2013, I2CVB, PROMISE12) was used. Of these, 109 scans were allocated for training and 6 (stratified by difficulty) for testing.

For both datasets, the segmentation used the nnUNet framework (2D UNet, default planner) with 5-fold cross-validation (100 epochs per fold) implemented in Jupyter Notebook and trained on an NVIDIA RTX 3060 GPU.

### 2.2 Definition of Medical Similarity Index

The Medical Similarity Index (MSI) addresses limitations of traditional segmentation metrics, including their inability to reflect local contour differences and lack of clinical interpretability. MSI, defined by bidirectional local distance (Kim et al. [14]), evaluates similarity between two discrete contours: the reference (gold standard) and the test. Each test point is paired with the reference using bidirectional local distance (BLD), and the final MSI is the average over all test points. A patient-level average MSI was also reported (see Supplementary Materials S1). Processing in PyCharm averaged 2.50 ± 0.74 s and 274.9 ± 19.9 MB (n = 10). MSI was computed on 2D slice contours. In MRI, 3D CNNs work well for near-isotropic volumes and structures with strong continuity, but inter-slice inconsistency from slice thickness, motion, or protocol can result in 3D models learning spurious continuity; in such cases, 2D or 2.5D approaches may be more robust [15], [16], [17].

### 2.3 Traditional metrics for evaluating segmentation

The demonstrated pipeline also included the calculation of traditional image segmentation metrics: Dice score, Jaccard score, average Hausdorff distance, and HD95 (the 95th percentile of the Hausdorff distance). We implemented each metric, including MSI, using a deterministic approach. All contour points were derived directly from segmentation masks, with no subsampling or resampling performed. The number of points in our calculations did not justify the use of randomized implementations, such as those in SciPy [18]. Surface Dice score [19] and Added Path Length (APL) [20] were calculated using open-access packages. For the prostate, the tolerance for surface Dice score was set to 3 mm, as in [21]. Dice and Jaccard scores quantify geometric overlap between segmentations. Hausdorff distance captures the maximum Euclidean distance between nearest neighbors. Added path length measures the total contour length required to match the reference contour [22].

### 2.4 Practical issues with contours: contour pairing, mask splitting

In medical image segmentation, some applications involve a single contour per slice (e.g., prostate). Others, such as fibroid segmentation, can contain multiple contours that must be correctly paired between reference and test masks for valid metric calculations. We implemented a center-of-mass (COM) matching strategy by assigning each reference COM to its nearest test COM. Ambiguous cases are flagged for manual review (as in Fig. S3). Unlike in general object detection, overlapping masks are not present in medical imaging. However, adjacent contours may touch; our pipeline initially treats them as a single mask but requires subsequent splitting. Candidate masks for splitting are identified using a minimum area threshold. They are then evaluated by the ratio of mask area to convex hull area (default threshold: 1.2). Concave masks exceeding this threshold are split along maximal convexity defect points, with cut placement determined by predefined parameters (see Algorithm 1.).

## 3 Results

**Fig. 1:**
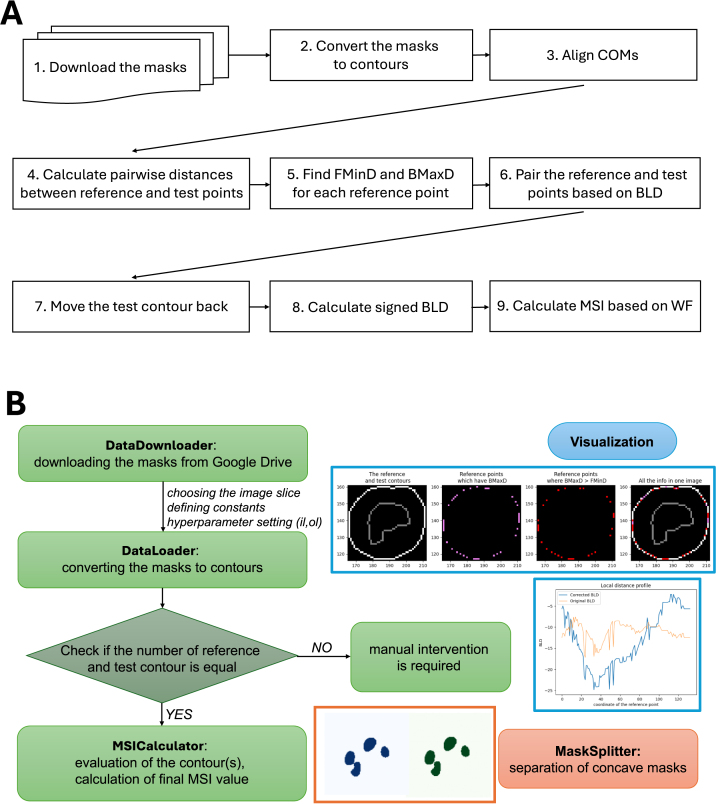
Medical Similarity Index (MSI) evaluation pipeline. **Panel A.** Workflow of MSI computation from segmentation masks to the final similarity score. Masks are converted to contours, aligned by centers of mass, and evaluated using bidirectional local distances, which are aggregated into the MSI via a weighting function. **Panel B.** Modular implementation of the automated pipeline, including data handling, contour evaluation, optional mask splitting for concave structures, visualization, and manual intervention when reference and test contours are inconsistent.

The provided Google Colaboratory notebook offers a user-friendly tutorial that guides users through MSI calculation, visualization, and experimentation (see Supplementary Materials S6). We demonstrated the pipeline using pelvic MRI fibroid segmentation masks that varied in shape and exhibited substantial segmentation errors. To emphasize the pipeline’s adaptability, we intentionally used a non–fine–tuned neural network to generate a wide range of segmentation errors. The calculation followed the workflow shown in Fig. 1 Panel A. An attached Colaboratory notebook provides the user with an opportunity to evaluate image masks. Although our demonstrations used only MRI-based segmentation masks, the pipeline operates solely on segmentation masks and does not use image intensity information. The outline of the evaluation steps is given in Fig. 1 Panel B. The pipeline incorporated procedures to handle problematic slices. Contour pairing was performed using a nearest COM assignment strategy; however, certain configurations may not be adequately resolved by this method. In addition, pairing errors could occur when two masks are in contact (Fig. S6). In such cases, accurate pairing can be restored by applying the mask-splitting algorithm, described in detail in the Supplementary Materials S5.

**Fig. 2:**
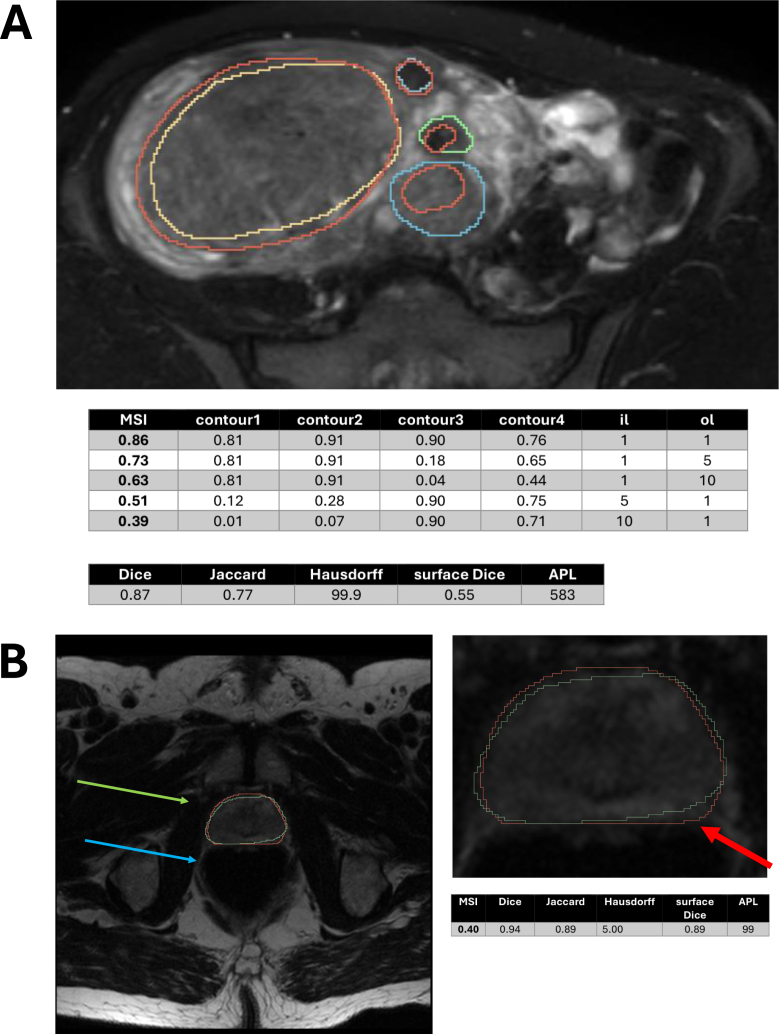
Comparison of MSI with conventional segmentation metrics: fibroid (A) and prostate (B) images. **Panel A** The reference contours are shown with purple (contour 1), green (contour 2), yellow (contour 3), and blue (contour 4) colors; the test contour is shown in red. The traditional metric values are also indicated. The MSI values were calculated with il=1,5,10 and ol=1,5,10 hyperparameters. **Panel B** The reference segmentation is shown in green, the neural network predicted segmentation is shown in red. Representative image where conventional overlap and distance-based metrics yield similar scores despite clinically relevant local differences, highlighted by MSI. The urinary bladder is indicated by the green arrow, and the prostate is indicated by the blue arrow in the T2 W MRI images.

The MSI can adapt to different clinical applications by modifying the il and ol hyperparameters. A representative example is shown in Fig. 2 Panel A. The MSI was calculated with default il=1, ol=1 hyperparameters, as well as with il=5,10 and ol=5,10. The traditional metric values were also indicated. If there are no special needs, a value of 0.86 could be clinically relevant, consistent with traditional metric values. In contrast, if we wanted to measure the volumes of the fibroids for treatment, the inner deviation would have more severe consequences. That is why the segmentation of this slice should have a low score (0.51 or 0.39). If the outer deviation had more clinical impact, an MSI value of 0.73 or 0.63 could be achieved. The MSI values with ol=5,10 are still larger than the MSI values with il=5,10, because the segmentation has more inner alterations than outer.

To demonstrate the pipeline’s clinical adaptability, a prostateanatomic segmentation dataset was selected. The impact of the outer deviation is crucial in current clinical applications, which is why the desired metric should reflect segmentation defects outside the reference segmentation. This phenomenon can be nicely studied in the following case. In Fig. 2 Panel B, we represented one slice of one test segmentation, where the outer segmentation deviation reached the urinary bladder. In this case, the segmentation is unacceptable. The traditional metrics and the MSI value with il=1 and ol=10 were calculated. While the traditional metrics fail to identify the extremely incorrect segmentation - Dice (0.94), Jaccard (0.89), surface Dice (0.89), APL (99 px) show high values, Hausdorff distance (5.0 mm) indicates medium value (mean ± SD: 6.80 ± 3.40) - the MSI (0.40), showing low value, characterizes the segmentation correctly. The metric values considering all slices and the masks are presented in the Appendix (see Fig. S10).

A sensitivity analysis showed significantly lower MSI for slice widths ≥ 3.3 mm than for ≤ 3.0 mm, whereas slice thickness had no significant effect on patient MSI. Details are provided in Supplementary Materials S4.

## 4 Discussion

We have developed an easy-to-use, adaptable pipeline for medical segmentation tasks. The pipeline facilitated comprehensive performance analysis by computing traditional segmentation metrics and introducing the Medical Similarity Index to assess segmentation agreement, thereby enhancing clinical relevance. Hyperparameters, such as il and ol levels, can be optimized based on clinical priorities, e.g., whether inside or outside deviations are more significant. We provided an outline for each image processing step. The code is available and follows sustainable, object-oriented design principles. This allows for straightforward customization and extension to suit specific research or clinical needs.

Although there are some reviews addressing evaluation metrics for general medical image segmentation [23], [24], as well as for special application areas such as blood vessel segmentation [25], retinal optical coherence tomography [26] and radiotherapy [27], currently standardized and clinically relevant evaluation protocols are not available. The evaluation of segmentation algorithms impacts their optimization, as it directly influences how performance is measured and compared [28]. However, there is no reliable method for assessing model performance, and statistical bias is also reported, likely due to incorrect metric implementation or usage [24]. Previous studies have shown that common quantification metrics do not reflect clinical acceptance in heart contouring from CT images [29]. Determining the most appropriate evaluation measures is a very challenging task; hence, it is essential to match the available metrics to the segmentation objectives [30].

The current work has some limitations. Our institution’s fibroid dataset was segmented by radiologists; thus, segmentation priorities and practices may differ between radiologists and radiotherapy planners, potentially influencing the resulting annotations. The proposed pipeline accepts only NIfTI-formatted images and masks, requiring conversion for other formats. Multi-mask images pose challenges for contour pairing; slices with unequal reference and test masks, or unresolved pairings using the closest-center-of-mass method (Fig. S3), are flagged for manual review. While this approach provides a functional baseline, more advanced pairing techniques could improve robustness, particularly for complex segmentations, and should be tailored to the intended clinical application. Furthermore, each contour yields an MSI value, which is aggregated per slice using the median, though alternative aggregation methods could be explored. Additionally, the pipeline does not support contour-specific weighting; however, splitting the segmentation into separate files, each containing only one contour, could address this challenge. Furthermore, while extension to 3D is feasible, anisotropic voxel spacing in reconstructed medical images may introduce bias. Also, computing pairwise distances in 3D may not be computationally feasible; hence, a 2D approach was adopted. However, the extension to 3D can be implemented using the currently available code.

In summary, we introduced an adaptable pipeline for evaluating medical image segmentation that combines established metrics with the proposed Medical Similarity Index to better reflect clinical priorities. The framework provides a transparent and extensible tool for reproducible performance assessment, and future work will focus on validating it across diverse datasets and clinical applications.

## CRediT authorship contribution statement

**Szuzina Fazekas:** Conceptualization, Data curation, Methodology, Visualization, Writing – original draft, Investigation, Software. **Bettina K. Budai:** Writing – review & editing, Conceptualization, Data curation. **Viktor Bérczi:** Resources, Writing – review & editing. **Pál Maurovich Horvat:** Resources, Writing – review & editing. **Zsolt Vizi:** Conceptualization, Supervision, Writing – review & editing, Methodology, Software.

## Ethics approval and consent to participate

This study was approved by the Institutional Review Board (Semmelweis University Regional and Institutional Committee of Science and Research Ethics, SE-RKEB: 172/2022). As this was a retrospective study, the need for written informed patient consent was waived by the ethics committee. All procedures performed in this study involving human participants were in accordance with the ethical standards of the Declaration of Helsinki. All patient data were analyzed anonymously.

## Declaration of generative AI

During the preparation of this work the authors used OpenAI ChatGPT in order to improve text quality. After using this tool, the authors reviewed and edited the content as needed and take full responsibility for the content of the publication.

## Funding

The research reported here was supported by the National Research, Development and Innovation Office (NKFIH) in Hungary [grant number RRF-2.3.1-21-2022-00006]. Szuzina Fazekas receives a grant from the Gedeon Richter Talentum Foundation within the framework of the Gedeon Richter Excellence PhD Scholarship.

## Declaration of competing interest

The authors declare that they have no known competing financial interests or personal relationships that could have appeared to influence the work reported in this paper.

## Data Availability

The codes and notebooks used in the current study are available in the cited GitHub repository [13]. The prostate dataset is available from the cited publication, the images of fibroid dataset are not publicly available due to personal rights, however, the generated segmentation masks are available from the cited repository.

## Appendix A Supplementary data

MMC S1 Supplementary materials provide detailed MSI definitions, algorithms, datasets, sensitivity analysis, and code resources. MMC S1
