# Supplementary material for: A clinically informed automated evaluation pipeline for medical image segmentation based on Medical Similarity Index
Source: Phys Imaging Radiat Oncol. 2026 Mar 17;38:100950. doi: 10.1016/j.phro.2026.100950 (PMC13068865; doi:10.1016/j.phro.2026.100950)
Supplement: MMC S1 — Supplementary materials provide detailed MSI definitions, algorithms, datasets, sensitivity analysis, and code resources. [file mmc1.pdf]

Supplementary materials

S1 Detailed definition of Medical Similarity Index

Firstly, we define the minimal distance between a point $\boldsymbol{p}$ and a contour C (discrete point set) as

$$\mathrm{d}_{\min}(\boldsymbol{p}, C) = \min_{\boldsymbol{q} \in C} \|\boldsymbol{p} - \boldsymbol{q}\|_2. \tag{1}$$

For calculating the *forward minimum distance* (FMinD), the closest point of the reference contour from a given test point must be found, i.e.

$$\mathrm{FMinD}(\boldsymbol{p}_{\mathrm{test}}, R) = \mathrm{d}_{\min}(\boldsymbol{p}_{\mathrm{test}}, R). \tag{2}$$

For computing the *backward maximum distance* (BMaxD), we iterate through all the reference points and find the closest test points for each. If there exists a reference point for which the endpoint of this distance is the previously selected test point, then the maximum of these distances will be chosen as BMaxD, i.e. for the reference contour R and a test point $\boldsymbol{p}_{\mathrm{test}}$, we have:

$$\mathrm{BMaxD}(R, \boldsymbol{p}_{\mathrm{test}}) = \max_{\boldsymbol{p}_{\mathrm{r}} \in R} \Big\{ \mathrm{d}_{\min}(\boldsymbol{p}_{\mathrm{r}}, T) : \|\boldsymbol{p}_{\mathrm{r}} - \boldsymbol{p}_{\mathrm{test}}\|_2 = \mathrm{d}_{\min}(\boldsymbol{p}_{\mathrm{r}}, T) \Big\}. \tag{3}$$

The BLD (corresponding to one test point) is the maximum of FMinD and BMaxD, for a point $\boldsymbol{p}$ and a contour R:

$$\mathrm{BLD}(\boldsymbol{p}_{\mathrm{test}}, R) = \max \Big\{ \mathrm{FMinD}(\boldsymbol{p}_{\mathrm{test}}, R), \mathrm{BMaxD}(R, \boldsymbol{p}_{\mathrm{test}}) \Big\}. \tag{4}$$

The *signed* BLD is negative if the test point is inside the reference contour and positive if the point is outside, indicated as $\mathrm{BLD}^{\pm}$.

The *Medical Similarity Index* (shortly MSI) is calculated based on a modified Gaussian curve, we call it the *Weight Function*, denoted by $\mathrm{WF}(d, \mathtt{l})$. Different *Weight Function* curves are demonstrated in Fig. S1, using different `l` values.

$$\mathrm{WF}(d, \mathtt{l}) = \exp\left(- \frac{d^2}{2 \cdot (10/\mathtt{l})^2} \right). \tag{5}$$

The value `l` is a user-defined constant, which can also have different values with respect to the test point is either inside or outside the reference contour. This penalty level can differentiate between the inside and outside test contour deviation, so the test contour's inside or outside alteration can be scored differently.

The score is calculated with the value `il` if the test point is inside the reference contour:

$$\mathrm{MCF}_{\mathrm{i}}(\boldsymbol{p}, R) = \mathrm{WF}(\mathrm{BLD}^{\pm}(\boldsymbol{p}, R), \mathtt{il}), \tag{6}$$

where `il` is pre-defined constant. The score is calculated with the value `ol` if the test point is outside the reference contour:

$$\mathrm{MCF}_{\mathrm{o}}(\boldsymbol{p}, R) = \mathrm{WF}(\mathrm{BLD}^{\pm}(\boldsymbol{p}, R), \mathtt{ol}), \tag{7}$$

where `ol` is pre-defined constant. The definition of the inside and outside penalty levels can reflect the particular needs of the clinical application. If the outside deviation has serious consequences in the current medical circumstances, i.e. a vital organ is close to the segmented lesion, the outside penalty can be higher. If the inside deviation is unacceptable for the current medical use, i.e. calculating the tumor volume for

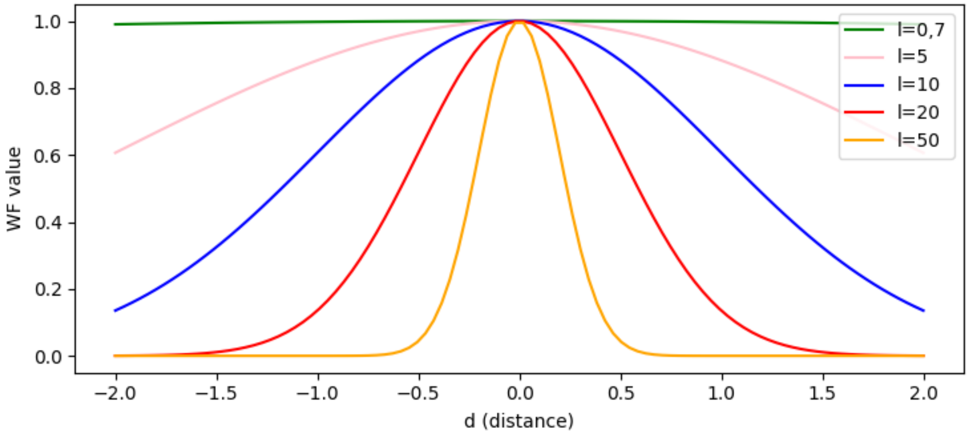

Figure S1: Weight Function. The $\mathrm{WF}(d, \mathtt{l})$ weight function with different fixed $\mathtt{l} = 0.7, 5, 10, 20, 50$ level values.

radiation therapy, the inside level can be set to a greater value. Using these user-defined constants in the MCF, a score is assigned to each test point. The final MSI value is the average of all scores along all test points. If the test point $\boldsymbol{p}$ is inside the reference contour, the $\mathrm{MCF_i}$ formula is used, if $\boldsymbol{p}$ is outside of the reference contour, $\mathrm{MCF_o}$ is used, with the following notations:

$$\mathrm{I}(R) = \{\boldsymbol{x} : \boldsymbol{x} \text{ is inside the reference contour}\} \tag{8}$$

and

$$\mathrm{O}(R) = \overline{\mathrm{I}(R)} \setminus R, \tag{9}$$

where $\overline{\mathrm{I}(R)}$ denotes the complement of $\mathrm{I}(R)$

The final MSI is defined by the following formula for a test contour T and a reference contour R:

$$\mathrm{MSI}(T, R) = \frac{1}{n}\left(\sum_{\boldsymbol{p}\in T\cap \mathrm{I}(R)} \mathrm{MCF_i}(\boldsymbol{p}, R) + \sum_{\boldsymbol{p}\in T\cap \mathrm{O}(R)} \mathrm{MCF_o}(\boldsymbol{p}, R)\right), \tag{10}$$

where $n = |T|$. Clearly, if the reference and test contours intersect, the MCF score of the intersection points is zero.

We additionally provided a patient-level MSI score, which allows the identification of exceptionally good or poor segmentations by summarizing slice-wise MSI values at the case level. The patient-level MSI score is calculated by the average of the MSI scores of the slices of the particular patient.

S2 Definitions of traditional metrics

An image can be defined as an $m \times n$ matrix. We define a mask as a group of pixels (i.e. matrix elements), which indicates the region of interest. We interpret a contour as the boundary of a mask, as clearly shown in Fig. S2.

One of the most commonly used image segmentation metrics is the *Dice index*.

$$D(M_R, M_T) = \frac{2 \cdot |M_R \cap M_T|}{|M_R| + |M_T|}, \tag{11}$$

where M_R is the reference mask, M_T is the test mask, $|M_R|$ is the (set) cardinality of the mask.

The Sorensen-Dice coefficient originates from statistics, where the similarity of two sets was assessed. The Dice score is also called *F1 score* and can be calculated as the harmonic mean of precision and recall.

$$\mathrm{F}_1(M_R, M_T) = \frac{2 \cdot \mathrm{TP}(M_R, M_T)}{2 \cdot \mathrm{TP}(M_R, M_T) + \mathrm{FP}(M_R, M_T) + \mathrm{FN}(M_R, M_T)} \tag{12}$$

where TP denotes the number of true positive, FP the false positive, FN false negative elements (or pixels) [1], where

- the true positive pixels mean the marked pixels which are correctly marked;
- the true negative pixels are the non-marked pixels in the ground truth segmentation which are not marked by the proposed segmentation;
- the false positive pixels are signed by the test segmentation, but correspond to the background on the reference segmentation.

The *Jaccard score* is also an area-based metric, which can be defined as the ratio of the number of elements of the intersection of two sets divided by the number of elements of the union of the two sets.

$$J(M_R, M_T) = \frac{|M_R \cap M_T|}{|M_R \cup M_T|} \tag{13}$$

In image processing Jaccard score is known as IoU (Intersection over Union).

The *Hausdorff distance* is a widely used metric in medical image analysis, which measures the largest segmentation error.

$$\mathrm{d}_H(M_R, M_T) = \max\left\{ \sup_{\boldsymbol{x} \in M_R} \mathrm{d}(\boldsymbol{x}, M_T), \sup_{\boldsymbol{y} \in M_T} \mathrm{d}(M_R, \boldsymbol{y}) \right\}, \tag{14}$$

where $\mathrm{d}(\boldsymbol{x}, M_T)$ is defined as $\mathrm{d}(\boldsymbol{x}, M_T) = \inf_{\boldsymbol{y} \in M_T} \mathrm{d}(\boldsymbol{x}, \boldsymbol{y})$

The directed average Hausdorff distance from point set X to point set Y can be calculated as the sum of all minimum distances from all points in X to Y divided by the number of points in X. The average Hausdorff distance is given by the average of the directed average Hausdorff distance from point set X to point set Y and from Y to X [2].

In our implementation, we paired the reference and test contours before the calculation of average Hausdorff distance. In some special cases it may differ from the usual implementation, but it is a more intuitive and logical approach. For each reference point the closest test point is selected, so if a test point of another contour is closer to the current reference point than the closest test point of its test contour pair, our implementation will use the latter one, while the usual implementations use the former one.

S3 Datasets

S3.1 Fibroid dataset

Patients who underwent uterus artery embolization in the Semmelweis University Medical Imaging Center between May 2016 and September 2020 were selected (overall 161 patients), and the pre-treatment baseline MR images were chosen. Overall, 31 patients were excluded, from which 10 patients' DICOM images were damaged, 16 patients had non-contourable fibroids (due to an extreme number of fibroids or non-identifiable fibroid boundary), and five patients were excluded because of the presence of adenomyosis.

All the MRI examinations were conducted at the Semmelweis University Medical Imaging Centre on 1.5 T equipment (Philips Ingenia 1.5T, Philips Healthcare, Best, Netherlands). A routine contrast-enhanced

pelvic MRI protocol was applied, which included T1W, T2W, T2W-SPAIR, and contrast-enhanced T1W-SPIR sequences. The imaging was executed with a 90° flip angle, 80 s echo time, 0.70 mm voxel spacing, and 400×400 reconstruction matrix.

The MRI images were exported from the institutional PACS (Picture Archiving and Communication System) in DICOM format and converted to NIfTI format. A radiology resident manually segmented the T2W sequence using 3DSlicer software (slicer.org), which were then validated by two expert radiologists with more than 10 years of experience in pelvic MRI imaging.

Out of the 130 patients, 124 were used for training, and 6 were selected for testing: 2 easy, 2 moderate, and 2 difficult cases. The easy cases (Fig. A, B) contained one or two fibroids with well-defined contours. The moderate cases (Fig. C, D) included either multiple fibroids or less distinct boundaries. The difficult cases (Fig. E, F) involved numerous fibroids with blurred or shaded contours, making delineation challenging. Representative slices for all six patients, along with the reference and predicted masks, are shown in Fig. S8.

The test segmentations were generated using the DKFZ `nnUNet` framework [3]. We created a `nnUNet` usage tutorial in the Google Colaboratory notebook, which guides users through all the necessary steps before preprocessing and training with the `nnUNet` framework. Only the Google Drive links of the training and testing zip files need to be provided. The `nnUNet` is widely used in medical imaging as it is an automated framework, allowing it to configure its hyperparameters based on the dataset fingerprint. The executable Google Colaboratory notebook for the neural network learning is also provided for reproducibility; however, we trained the neural network in local computer in PyCharm IDE. Specifically, we used 2D U-Net with the default planner for 5 folds, 100 epochs per fold, the initial learning rate was 0.01. The training was done on an NVidia GeForce RTX 3060 12GB Dual V2 OC video card; one epoch took about 100 seconds.

S3.2 Prostate dataset

An open-access multi-site dataset for prostate MRI segmentation was used for demonstration purposes with corresponding anatomical prostate segmentations [4]. Prostate segmentation on MRI images is a challenging task due to the heterogeneity of prostate structure. However, in the case of radiation therapy, precise prostate anatomic segmentation is crucial due to the proximity of the urinary bladder, which makes the evaluation very crucial. When radiation therapy is applied, any outer deviation of the segmentation mask can cause radiation damage to the urinary bladder. This damage will have a huge impact on living conditions, such as incontinence.

The dataset consists of a total of 115 prostate T2W MRI images and corresponding segmentation masks. The images are from six different data sources out of three public datasets (NCI-ISBI 2013 [5], I2CVB [6], PROMISE12 [7]). The preprocessing included conversion to NIfTI format, centering the prostate, and resizing to a size of 384×384 in the axial plane. Please see the imaging protocol details in S1.

From the 115 MRI scans, 109 were used for training and 6 were selected for testing: 2 easy, 2 moderate and 2 difficult cases. The training was done with nnUNet using the same Google Colaboratory notebook as in case of the fibroid dataset. The test segmentations were produced using the DKFZ `nnUNet` framework [3]. For reproducibility, the executable Google Colaboratory notebook is publicly available; however, the network training was performed locally using the PyCharm IDE. In particular, a 2D U-Net architecture with the default planner was employed, using 5-fold cross-validation and 100 training epochs per fold with an initial learning rate of 0.01. Training was conducted on an NVIDIA GeForce RTX 3060 12GB Dual V2 OC GPU, with an average training time of approximately 100 seconds per epoch.

S4 Sensitivity analysis of MSI

To assess the potential influence of slice width and slice thickness on the MSI metric, we conducted a sensitivity analysis with respect to these parameter. The analysis was performed using the training subset of the datasets, with predicted segmentations generated by the previously trained nnUNet model during inference. As the objective of this experiment was not to evaluate segmentation quality, but rather to investigate the robustness of MSI against variations in slice width and slice thickness, the use of training

Table S1: Details of the multi-site prostate dataset and corresponding imaging protocols.

Dataset	Institution	No. of cases	Field strength (T)	Resolution (in-/through-plane) (mm)	Manufacturer
1	RUNMC	30	3	0.60–0.625 / 3.6–4.0	Siemens
2	BMC	30	1.5	0.40 / 3.0	Philips
3	HCRUDB	19	3	0.67–0.79 / 1.25	Siemens
4	UCL	13	1.5, 3	0.325–0.625 / 3.0–3.6	Siemens
5	BIDMC	12	3	0.25 / 2.2–3.0	GE
6	HK	12	1.5	0.625 / 3.6	Siemens

Table S2: Comparison of dataset characteristics and neural network training parameters.

	Fibroid dataset	Prostate dataset
Institution	Semmelweis University	Open access
Number of cases	130	115
Modality	MRI	MRI
Slice thickness	4.0-5.0 mm	2.2-4.0 mm
Image acquisition	Philips 1.5T routine pelvic protocol	Various (see Table S1)
Segmentation method	2D nnUNet	2D nnUNet
Epochs per fold	100	100
Initial learning rate	0.01	0.01
Approx. seconds per epoch	100	100

cases does not introduce bias into the results.

As the fibroid dataset is from our Institute, the raw DICOM data was also available, so we could use the slice thickness as well. Levene's test indicated no significant differences in variance among slice-width groups ($W = 0.91$, $p = 0.49$), supporting the assumption of homogeneity of variances. A one-way ANOVA showed no significant effect of slice width on patient MSI ($F = 2.04$, $p = 0.066$). As for slice thickness, Levene's test indicated no significant differences in variance among slice-width groups ($W = 1.07$, $p = 0.36$) and one-way ANOVA showed no significant effect of slice width on patient MSI ($F = 1.75$, $p = 0.18$).

For the prostate dataset, only slice width data was available, so we carried out sensitivity analysis with respect to the z coordinate of voxel size. The mean and standard deviation of patient-level MSI scores for each slice width group are presented in Table S3. Levene's test indicated no significant differences in variance among slice-width groups ($W = 1.04$, $p = 0.41$), supporting the assumption of homogeneity of variances. A one-way ANOVA revealed a significant effect of slice width on patient MSI ($F = 14.71$, p ¡ 0.001). Post-hoc Tukey HSD tests identified significantly lower MSI values for slice widths ≥ 3.3 compared with ≤ 3.0.

Table S3: Patient MSI by slice width in the prostate dataset training cases.

Slice width	Mean MSI	SD	number of patients
1.25	0.87	0.03	18
2.20	0.87	0.02	5
2.80	0.88	0.03	4
3.00	0.85	0.04	32
3.30	0.80	0.05	8
3.60	0.79	0.04	36
4.00	0.79	0.04	7

Table S4: Patient-wise segmentation performance metrics. Regarding the fiboid dataset, the description of the six test cases are indicated. The number of slices with correct pairing indicates the number of slices where MSI could be automatically calculated. The number of nonempty slices includes every slice which has either reference or test mask (or both). The patient-level MSI scores and traditional metric values are also provided.

Patient number	Number of slices with correct pairing	Number of nonempty slices	Patient-level (average)			
			MSI	Dice	Jaccard	Hausdorff (px)
1	81	164	0.82	0.87	0.77	24.28
2	49	97	0.83	0.85	0.74	18.31
3	17	18	0.85	0.89	0.81	10.46
4	6	13	0.42	0.40	0.32	63.41
5	5	16	0.80	0.86	0.76	36.80
6	14	14	0.85	0.96	0.93	3.60

S5 The pipeline separates the problematic slices

If the number of reference and test masks are not equal or the contour pairing is not possible with the currently implemented closest COM method, the pipeline will require manual intervention. These problematic slices are separated by the pipeline. In the demonstration concerning fibroids, there are many problematic slices as the number of fibroids in one patient has a wide range (from 1 to 14 in our dataset), see an example on Fig. S4.

S5.1 Contour pairing

In some medical applications, a segmentation mask consists of only one segment (e.g., prostate anatomic segmentation). In other cases, such as the fibroids, there can be more than one contour on one image slice. In such cases, the reference and test contours must be paired for reasonable metric calculations — not only for MSI but also for average Hausdorff distance. In our implementation, we calculated the center of mass (COM) for each reference and test contours, and assign the closest test COM for each reference COM. The algorithm works properly on easy and difficult slices as well (see examples in Fig. S5). If there are more than one test COM assigned for a reference COM (as in Fig. S3), we consider the slice as a special case, which needs manual intervention.

S5.2 Mask splitting

In image recognition, object detection is a well known discipline with variety of publications and algorithms [8]. In this field, a commonly observed challenging task is to handle overlapping objects. In medical imaging, two lesions or organs can touch each other without overlap - there are no pixels which correspond to both objects. For this reason the general mask splitting algorithms cannot be used in our pipeline. In our segmentation masks, if two contours are touching, it means that some pixels of one contour is adjacent to some pixels of the other contour. In that case, the two contours are considered as one mask (consequently one contour), but the further calculations would need them as separate contours.

The mask splitting algorithm was prepared to solve the challenging cases where two masks are touching (see Fig. S6). If the contour pairing is not possible due to the different number of reference and test masks, mask splitting might be able to solve this problem. If there is one or more contours, which has common pixels (so they are touching) and they fulfill the criteria for the splitting algorithm, the separation of the previously mentioned contours enables the algorithm to pair the contours and the user can continue the evaluation with the pipeline.

For selecting the masks for splitting, it is possible to set a minimum mask area (`MIN_AREA`), only the masks above this threshold will be candidates for splitting. The used algorithm (see Algorithm 1.) separates the concave masks based on the ratio of the mask area and the convex hull area. A user defined threshold ratio is set (the default value is 1.2), only the masks above this ratio will be separated. The algorithm finds the

convexity defects of the concave masks, which can be defined as the maximal distance of the masks from the corresponding side of the convex hull. Based on further parameters, the cut will be applied along the maximal convexity defect points.

Algorithm 1: The steps of the mask splitting algorithm.

```
Calculate the area of the mask;
if area of the mask > MIN_AREA then
    Find the contour of the mask;
    Find the convex hull;
    if there are more than 2 convex hull points and area_ratio > 1.2 then
        Find the convexity defects;
        if length of convexity defect < 0.5 · max_length then
            Throw out
        end
        if only one convexity defect point is left then
            we do not discuss: 'horseshoe' shaped mask
        end
        if there are more convexity defect points left then
            the splitting is between the two closest ones
        end
    end
end
```

The OpenCV [9] package was used for the mask splitting algorithm, by name the `findContours`, `convexHull` and `convexityDefects` functions.

S6 Google Colaboratory notebook

The notebook's preparation part consists of the necessary imports and cloning of the GitHub repository. The URL of the reference and test files must be provided for the downloading process.

In the MSI calculation part, the testing calculations paragraph inputs the number of the current patient and current slice, and the inside and outside penalty levels can be declared; the algorithm will give one MSI value as the final result. The Visualization paragraph features various images and graphs to facilitate understanding and experimentation with the dataset and MSI values. The Split masks paragraph provides an opportunity to handle the concave mask case, where two touching masks are drawn together (see **??**).

S7 Figures

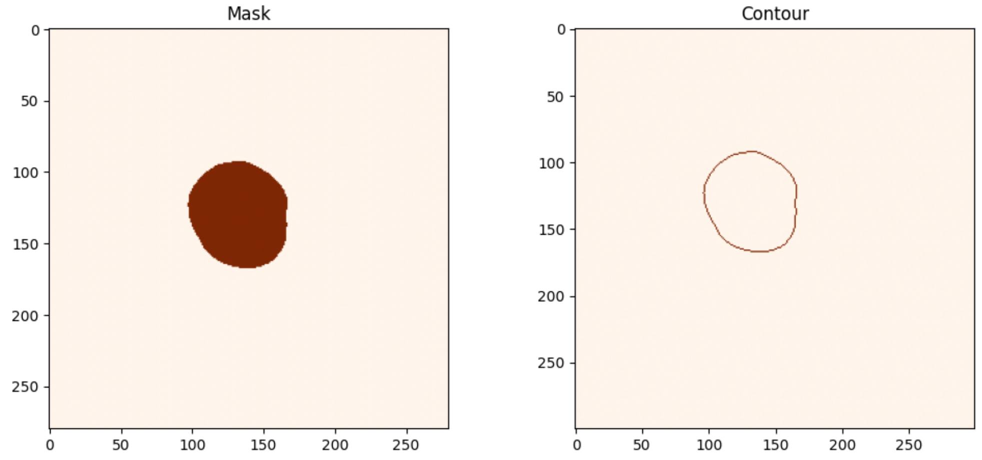

Figure S2: Definition of mask and contour. We define a mask as all the pixels corresponding to the segmented area, while we mean the boundary of the mask by the contour.

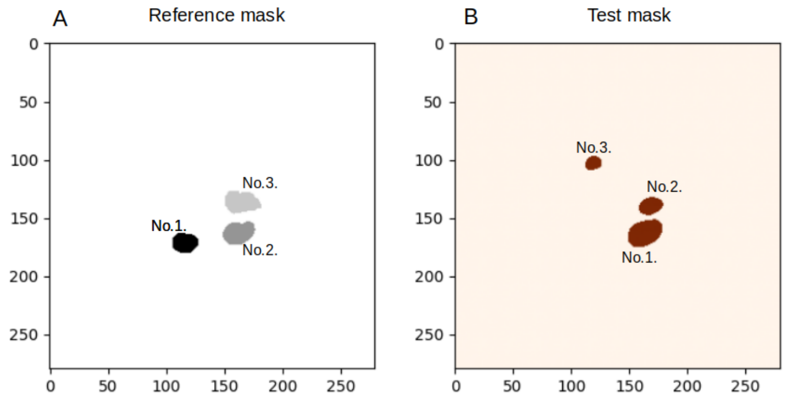

Figure S3: Representative slice for contour pairing. In this representative slice, the contour pairing with the closest center of mass method cannot be done. For reference contour no. 1, the closest test COM is test contour no. 1. For reference contour no. 2, the closest test COM is also test contour no. 1. For this reason the algorithm cannot handle this slice.

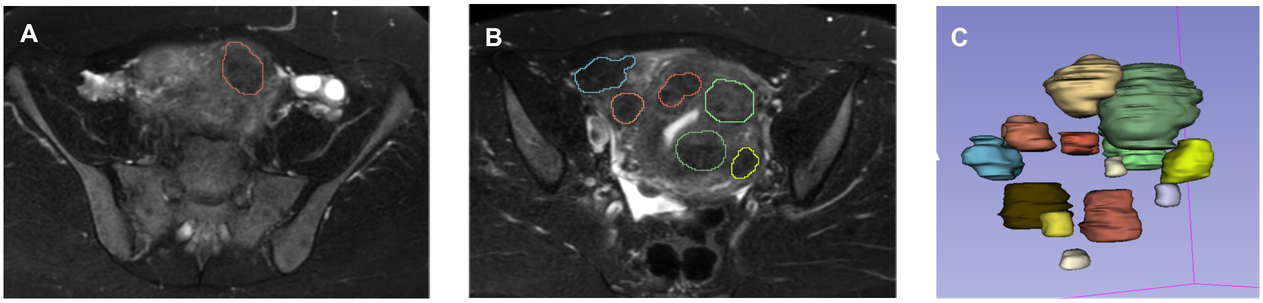

Figure S4: Representative slices of a patient with 14 fibroids. In our dataset, the number of fibroids in one patient can vary from 1 to 14. In case of the patient with 14 fibroids, the number of masks in one slice ranges from 1 to 6. In some slices (see Panel A.), there is only one mask, but other slices may interfere with more fibroids, thus there are slices with six masks as well (see Panel B.). The T2W SPAIR axial MRI images with the segmentation masks, as well as the 3D reconstruction of the masks are shown in the images.

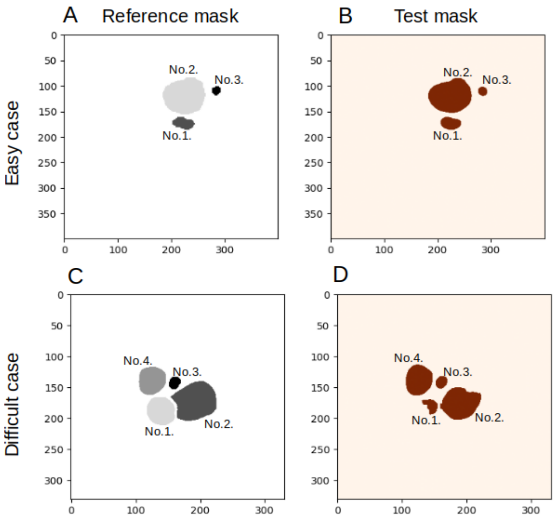

Figure S5: Examples for contour pairing algorithm. The contour pairing algorithm can pair easy and difficult cases as well. The reference masks (Panel A. and Panel C.) are colored by gray, the test masks (Panel B. and Panel D.) are colored by red.

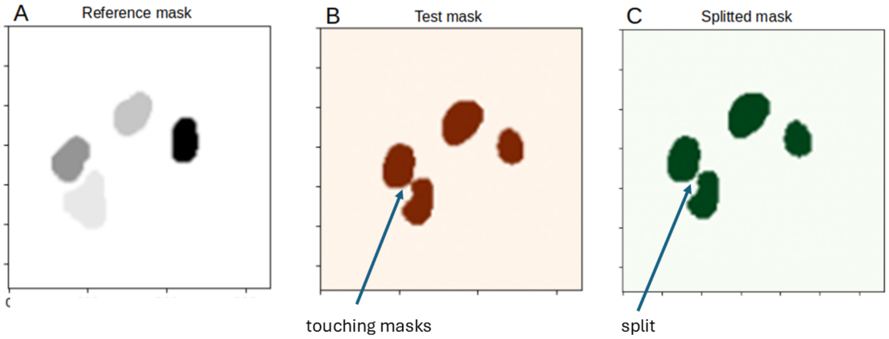

Figure S6: Representative slice for mask splitting. In this representative slice, there are four reference contours (Panel A.), while there is only three separate test contours (Panel B.), as two masks are touching. With the mask splitting algorithm, it is possible the separate the touching masks (Panel C.) and the automatic evaluation can be performed.

slice index	MSI	Dice	Jaccard	Hausdorff
6	0.21	0.92	0.86	5.39
7	0.40	0.94	0.89	5.00
8	0.42	0.95	0.91	5.66
9	0.69	0.95	0.90	8.49
10	0.73	0.96	0.93	4.00
11	0.62	0.98	0.96	3.00
12	0.73	0.94	0.88	9.00
13	0.60	0.97	0.95	3.61
14	0.77	0.97	0.94	5.39
15	0.67	0.95	0.91	7.28
16	0.26	0.92	0.86	7.07
17	0.73	0.96	0.92	4.24
18	0.63	0.90	0.81	13.89
19	0.55	0.84	0.72	5.83
20	0.57	0.05	0.03	14.21

Table S5: The MSI values for the slices shown in Fig. S10. The MSI values were calculated with `il` $= 1$, `ol` $= 10$ hyperparameters.

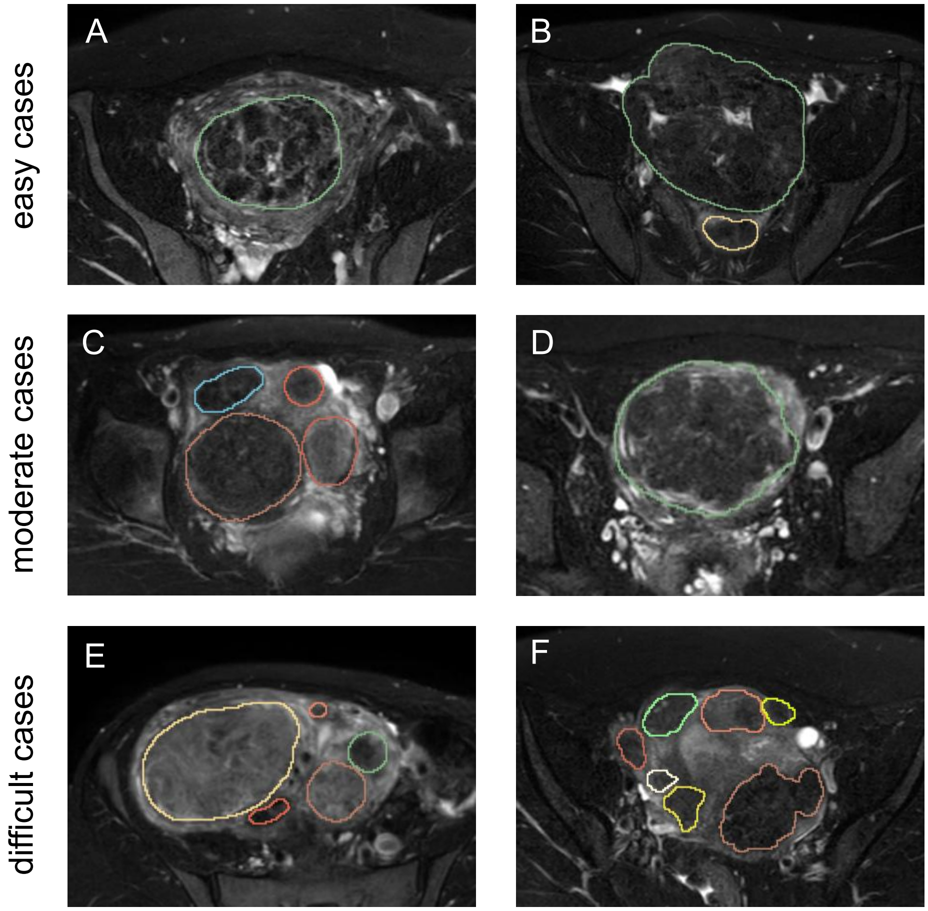

Figure S7: Test patients for fibroid segmentation neural network. For testing the fibroid segmentation neural network, six patients were selected from our dataset: two easy (Panel A and B), two moderate (Panel C and D) and two difficult cases (Panel E and F).The easy cases had a few fibroids with well defined contours, while the more difficult cases had lots of fibroids and blurred contours. The T2W SPAIR axial MRI images with the segmentation masks are shown in the images.

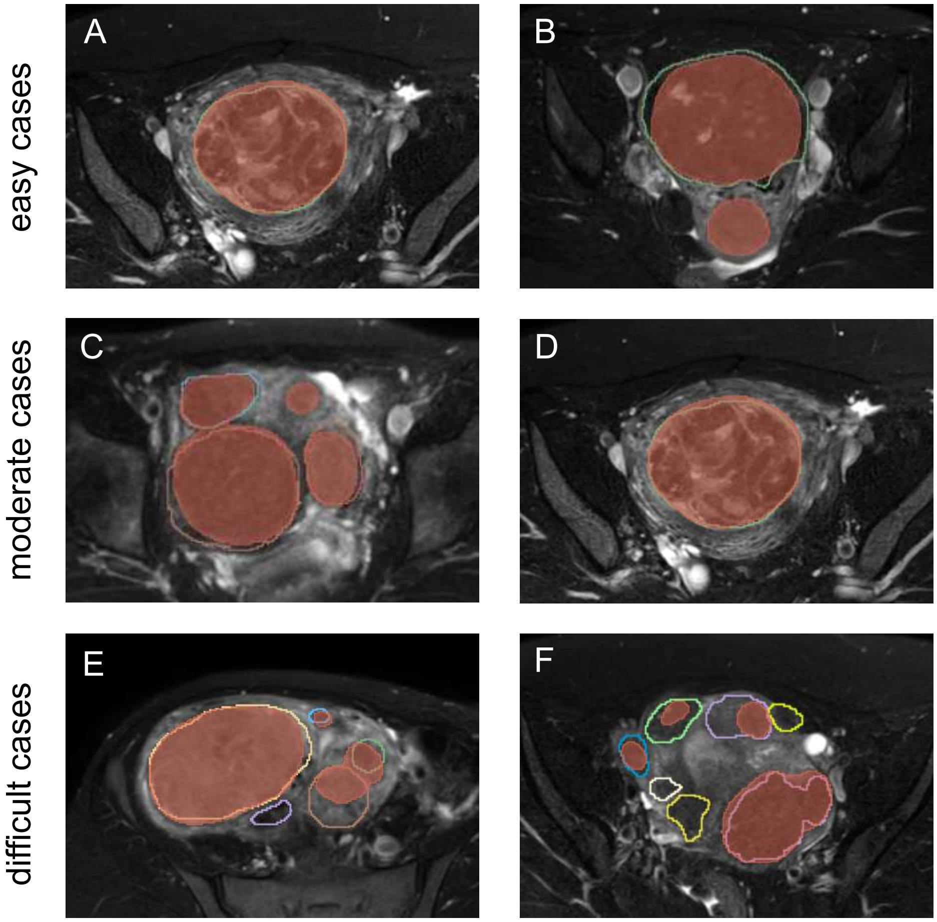

Figure S8: Result masks of the fibroid segmentation neural network. For testing the fibroid segmentation neural network, six patients were selected from our dataset: two easy (Panel A and B), two moderate (Panel C and D) and two difficult cases (Panel E and F). One slice of each patient is selected, the reference masks are show with contours, the test masks are shown in red filled areas. The T2W SPAIR axial MRI images with the segmentation masks are shown in the images.

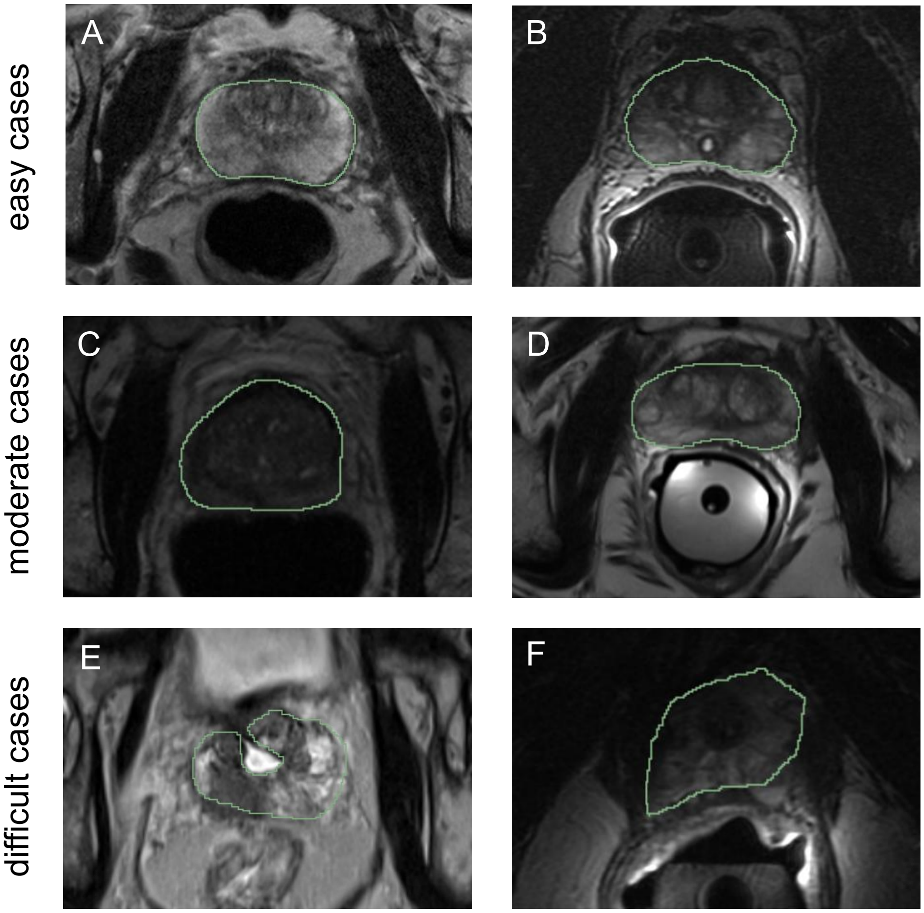

Figure S9: Test patients for prostate segmentation neural network. For testing the prostate segmentation neural network, six patients were selected from our dataset: two easy (Panel A and B), two moderate (Panel C and D) and two difficult cases (Panel E and F).The easy cases had well defined boundaries, while the more difficult cases had blurred contours, irregular shape or worse image quality. The T2W SPAIR axial MRI images with the segmentation masks are shown in the images.

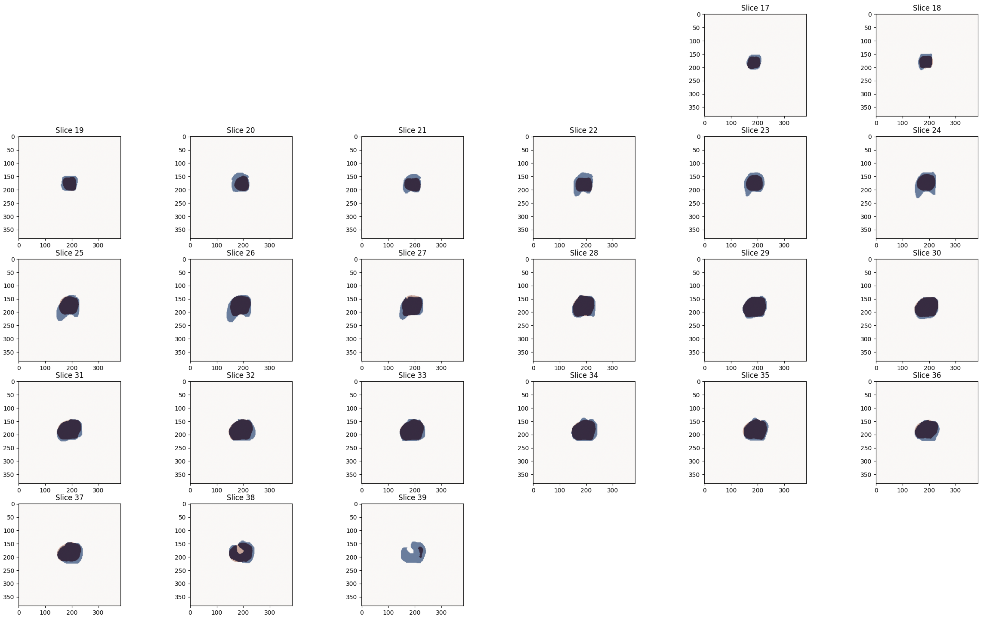

Figure S10: The segmentation masks of a representative patient. The reference masks are shown in blue, the neural network-proposed test masks are shown in orange. The MSI values for each slice were calculated with `il` = 1, `ol` = 10 hyperparameters, the values are shown in Table S5.

References

[1] Müller D, Soto-Rey I, Kramer F. Towards a guideline for evaluation metrics in medical image segmentation. BMC Res Notes. 2022;15(1). https://doi.org/10.1186/s13104-022-06096-y.

[2] Aydin OU, Taha AA, Hilbert A, Khalil AA, Galinovic I, Fiebach JB, et al. On the usage of average Hausdorff distance for segmentation performance assessment: hidden error when used for ranking. Eur Radiol Exp. 2021;5(1):4. https://doi.org/10.1186/s41747-020-00200-2.

[3] Isensee F, Jaeger PF, Kohl SAA, Petersen J, Maier-Hein KH. nnU-Net: a self-configuring method for deep learning-based biomedical image segmentation. Nat Methods. 2021;18:203-11. https://doi.org/10.1038/s41592-020-01008-z.

[4] Liu Q, Dou Q, Yu L, Heng PA. Semi-supervised medical image segmentation via uncertainty rectified pyramid consistency. Med Image Anal. 2022;78:102517. https://doi.org/10.1016/j.media.2022.102517.

[5] Bloch N, Madabhushi A, Huisman H, Freymann J, Kirby J, Grauer M, et al. NCI-ISBI 2013 challenge: automated segmentation of prostate structures. The Cancer Imaging Archive. 2015;370(6):5. https://doi.org/10.7937/K9/TCIA.2015.zF0vlOPv.

[6] Lemaître G, Martí R, Freixenet J, Vilanova JC, Walker PM, Meriaudeau F. Computer-aided detection and diagnosis for prostate cancer based on mono and multi-parametric MRI: a review. Comput Biol Med. 2015;60:8-31. https://doi.org/10.1016/j.compbiomed.2015.02.009.

[7] Litjens G, Toth R, van de Ven W, Hoeks C, Kerkstra S, van Ginneken B, et al. Evaluation of prostate segmentation algorithms for MRI: The PROMISE12 challenge. Med Image Anal. 2014;18(2):359-73. Available from: https://www.sciencedirect.com/science/article/pii/S1361841513001734. https://doi.org/https://doi.org/10.1016/j.media.2013.12.002.

[8] Amit Y, Felzenszwalb P, Girshick R. Object detection. In: Computer Vision: A Reference Guide. Springer; 2021. p. 875-83. https://doi.org/10.1007/978-3-030-63416-2_660.

[9] OpenCV. OpenCV: Open Source Computer Vision Library; 2024. Accessed: 2026-03-08. https://opencv.org/about/.
